# Identification of Malaria-Selective Proteasome β5 Inhibitors Through Pharmacophore Modeling, Molecular Docking, and Molecular Dynamics Simulation

**DOI:** 10.3390/ijms252211881

**Published:** 2024-11-05

**Authors:** Muhammad Yasir, Jinyoung Park, Eun-Taek Han, Jin-Hee Han, Won Sun Park, Wanjoo Chun

**Affiliations:** 1 Department of Pharmacology, Kangwon National University School of Medicine, Chuncheon 24341, Republic of Korea; yasir.khokhar1999@gmail.com (M.Y.); jinyoung0326@kangwon.ac.kr (J.P.); 2 Department of Medical Environmental Biology and Tropical Medicine, Kangwon National University School of Medicine, Chuncheon 24341, Republic of Korea; ethan@kangwon.ac.kr (E.-T.H.); han.han@kangwon.ac.kr (J.-H.H.); 3 Department of Physiology, Kangwon National University School of Medicine, Chuncheon 24341, Republic of Korea; parkws@kangwon.ac.kr

**Keywords:** *P. falciparum*, proteasome, pharmacophore, molecular docking, molecular dynamics simulation, FDA drugs, malaria

## Abstract

Malaria remains a global health challenge, with increasing resistance to frontline antimalarial treatments such as artemisinin (ART) threatening the efficacy of current therapies. In this study, we investigated the potential of FDA-approved drugs to selectively inhibit the malarial proteasome, a novel target for antimalarial drug development. By leveraging pharmacophore modeling, molecular docking, molecular dynamics (MD) simulations, and binding free-energy calculations, we screened a library of compounds to identify inhibitors selective for the Plasmodium proteasome over the human proteasome. Our results highlighted Argatroban, LM-3632, Atazanavir Sulfate, and Pemetrexed Hydrate as promising candidates, with Argatroban and Pemetrexed Hydrate showing the highest binding affinity and selectivity toward the malarial proteasome. MD simulation and gmx_MMPBSA analysis confirmed the compounds’ ability to remain within the active site of the malarial proteasome, while some exited or exhibited reduced stability within the human proteasome. This study underscores the potential of proteasome-targeting drugs for overcoming malarial drug resistance and paves the way for the further optimization of these compounds.

## 1. Introduction

Malaria continues to pose a significant global health threat, placing over a third of the world’s population at risk [1,2]. This infectious disease, caused by protozoan parasites from the *Plasmodium* genus, has been a persistent challenge for thousands of years [3,4,5]. Despite the development of numerous antimalarial drugs, starting with the discovery of quinine, *Plasmodium* parasites have demonstrated an alarming ability to develop resistance to many available treatments, including artemisinin (ART) and its derivatives, which form the cornerstone of first-line therapy for malaria [6,7]. ART resistance has become widespread in many regions and has also emerged independently in Sub-Saharan Africa [8,9,10]. Research has linked this resistance to mutations in the *Kelch13* gene, indicating a convergent evolutionary response in the parasite across these regions [11,12]. The potential for ART resistance to expand beyond its current manifestation in the ring stage of the parasite’s life cycle raises significant concerns for global health, with the risk of widespread treatment failure echoing the public health crisis that occurred in the late 20th century, when *Plasmodium* parasites developed resistance to chloroquine [13,14,15,16].

Considering their growing drug resistance, targeting the proteasome of *Plasmodium* parasites has gained attention as a promising strategy for antimalarial drug development [17,18,19]. The proteasome is a large multi-subunit protein complex composed of a 20S proteolytic core and regulatory components that control the entry of proteins marked for degradation [20]. The 20S core consists of two outer α-subunit rings and two inner β-subunit rings, with only the β1, β2, and β5 subunits exhibiting catalytic activity. β1 shows caspase-like activity, while β2 has trypsin-like activity, and β5 demonstrates chymotrypsin-like activity [21]. Inactivation of the β5 subunit causes significant phenotypic changes, highlighting its critical role [22]. The proteasome regulates protein turnover, misfolded protein degradation, and biological pathways [22]. Proteasome inhibitors induce cell cycle arrest and apoptosis in tumor cells, leading to their use in multiple-myeloma treatment [23]. Proteasome inhibitors also exhibit efficacy against parasitic organisms like *Mycobacterium tuberculosis*, *Trypanosoma brucei*, and *Plasmodium* spp. [24,25]. Pathogen proteasomes have emerged as viable targets for antimicrobial drugs, as demonstrated in preclinical studies focused on *Leishmania* and *Trypanosoma* parasites [26]. These inhibitors block *Plasmodium falciparum* replication in various life cycle stages, making the proteasome a promising drug target [27,28]. However, progress in animal studies is hindered by toxicity due to cross-reactivity with the host proteasome.

This study focuses on identifying selective inhibitors of the malarial proteasome β5 subunit from a library of FDA-approved drugs, using a combination of different approaches including pharmacophore modeling, molecular docking, molecular dynamics (MD) simulations, and gmx_MMPBSA analysis. By comparing the binding affinities and stability of interactions for both human and malarial proteasomes, this research aims to pinpoint compounds that offer high selectivity for the parasite’s proteasome, potentially paving the way for more effective and safer antimalarial treatments.

## 2. Results

### 2.1. Receptor–Ligand Interaction Pharmacophores

**Figure 1:**
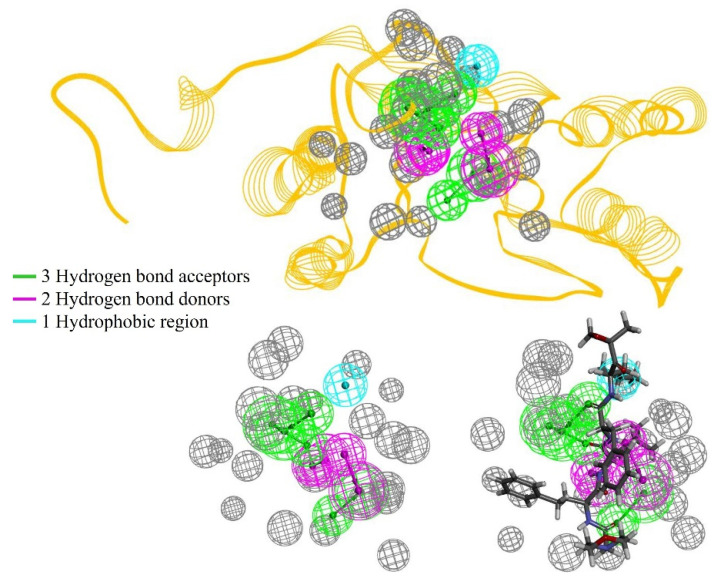
The interaction pharmacophore model of malarial proteasome and carfilzomib.

One of the challenging parts of drug discovery is to tackle the side effects of a compound’s non-selective action. To generate pharmacophore features of the receptor–ligand interaction, the malarial proteasome β5 subunit bound to carfilzomib was utilized, and the RLIPG (Receptor–Ligand Interaction Pharmacophore Generation) approach of Discovery Studio was applied. The proteasome β5 subunit was accessed from PDB ID: 7LXT and docked with carfilzomib. Carfilzomib was selected for this study due to its FDA approval as a treatment for refractory or relapsed multiple myeloma. Additionally, it has been shown to effectively kill the asexual blood stage of *P. falciparum* [29] and exhibits strong synergy with artemisinin [30]. In addition, analogs of carfilzomib have also demonstrated oral bioavailability in previous studies [31] (Figure 1). However, the dosage of carfilzomib needed for effective malaria treatment would be harmful to host cells [32,33].

The pharmacophore model features include specific hydrogen-bond acceptors and donors. These elements contribute to selective interactions with regions that exclude volume. Therefore, the selected top pharmacophore model includes three hydrogen-bond acceptors (represented in green), two hydrogen-bond donors (shown in magenta), and a hydrophobic region (in cyan), with the excluded volume areas marked in gray.

### 2.2. Library Screening

An FDA-approved drug library with a unique collection of 3153 compounds for high-throughput screening (HTS) and high-content screening (HCS) was accessed, and the library screen approach of Discovery Studio was employed to screen 344 compounds from the library, with fit values ranging from 3.4750 to 4.2011 × 10^−7^. The ligand Neotame manifested the highest fit value of 3.4750, while the ligand Asunaprevir demonstrated the lowest fit value of 4.2011 × 10^−7^.

### 2.3. Molecular Docking

The CDocker module of Discovery Studio was employed for the molecular docking studies and the prediction of negative docking energy values, which included both the CDocker energy and the CDocker interaction energy. CDocker energy represents the overall docking energy, considering the 3D structure and physicochemical properties of both the ligand and the protein. In contrast, CDocker interaction energy focuses on the energy associated with the interactions between the ligand and receptor, accounting for intermolecular forces such as hydrogen bonding, electrostatic interactions, and van der Waals forces [34,35].

All the screened compounds were docked against both human and malarial proteasomes and were sorted based on their selectivity toward the malarial proteasome. Therefore, from the 344 pharmacophore screened compounds, 47 compounds were chosen that manifested low docking energies to the malarial proteasome compared with the human proteasome (Supplementary Data Table S1). Of the 47 compounds, the top 15 compounds are depicted in Table 1.

Among the listed top 15 compounds, Difelikefalin emerged as the standout candidate, exhibiting a CDocker energy of −91.3408 kcal/mol for the malarial proteasome compared with −76.1867 kcal/mol for the human proteasome, highlighting its potential as a highly selective inhibitor in the molecular docking analysis. LM-3632 also showed promising results, with a CDocker energy of −73.1960 kcal/mol for the malarial versus −64.4805 kcal/mol for the human proteasome, suggesting a favorable binding preference toward the malarial target. Ritonavir and Atazanavir Sulfate demonstrated moderate selectivity as well, with CDocker energies of −67.7914 kcal/mol and −64.1737 kcal/mol for the malarial proteasome, respectively, compared with −61.2586 kcal/mol and −58.6843 kcal/mol for the human proteasome.

Additionally, Fosamprenavir displayed binding energies of −59.9645 kcal/mol for the malarial and −56.2256 kcal/mol for the human proteasome, indicating a moderate selectivity. Quinapril Hydrochloride and Zofenopril Calcium also showed promising profiles, with CDocker energies of −47.5670 kcal/mol and −44.3744 kcal/mol for the malarial versus −43.8536 kcal/mol and −36.7981 kcal/mol for the human proteasome, respectively. While these compounds demonstrate potential for selective inhibition, their lower absolute energy values compared with the top candidates suggest that they may require further analysis to see their effectiveness.

Elagolix Sodium is particularly noteworthy, as it presented a large disparity between the binding energies: −39.4056 kcal/mol for the malarial proteasome compared with a much less favorable −24.1487 kcal/mol for the human proteasome, indicating a strong potential for malaria selectivity despite its relatively lower absolute binding strength.

### 2.4. Binding Interaction Analysis of Screened Enamine Compounds

The data provides a detailed comparison of the receptor–ligand interactions between several FDA-approved drugs and both the malarial and the human proteasome. The bond distances between the key amino acid residues in each proteasome and the drug molecules were analyzed to assess selectivity and binding strength, with a focus on identifying compounds that exhibit stronger interactions with the malarial proteasome. Salt bridges (bold), highlighted by certain amino acid interactions, play a crucial role in stabilizing these drug–protein complexes (Table 2).

Difelikefalin demonstrated more interactions with the malarial proteasome, particularly with Gly129, Thr1, Gly47 and Ser21, with bond distances ranging from 2.57 Å to 2.99 Å. In contrast, its interactions with the human proteasome, such as with Thr21 and Gly47, showed slightly shorter bond distances but fewer interaction points. The drug also showed two salt-bridge formations against the malarial proteasome, suggesting that Difelikefalin may be more selective toward the malarial proteasome. Fosamprenavir showed similar but slightly weaker interactions with the human proteasome.

LM-3632 also demonstrated favorable selectivity for the malarial proteasome, with multiple interactions, including with Gly23, Gly47, Ser21, and Ala49, with bond distances ranging from 2.01 Å to 3.09 Å. In the human proteasome, LM-3632 binds to Gly23, Ser21, Gly47, and Met45 with similar bond lengths but fewer interaction sites, indicating a preference for the malarial target.

Atazanavir Sulfate showed strong binding interactions with the malarial proteasome, particularly with Ser21 and Thr1, with bond distances as short as 1.89 Å. In the human proteasome, it interacted with Thr21, Lys33, Thr1, and Ala49 with slightly longer bond distances, suggesting better binding affinity and selectivity for the malarial proteasome. Although it has fewer sites, it binds closely.

Quinapril Hydrochloride demonstrated interactions involving Thr1, Gly47, and Ala49, forming bonds as short as 1.98 Å. However, it also interacted with key residues in the human proteasome, including Gly23, Thr21, and Lys33. Ritonavir displayed comparable interactions with both proteasomes but the bond with Asp116, forming a salt bridge in the malarial proteasome, suggests enhanced selectivity to the malarial proteasome.

**Figure 2:**
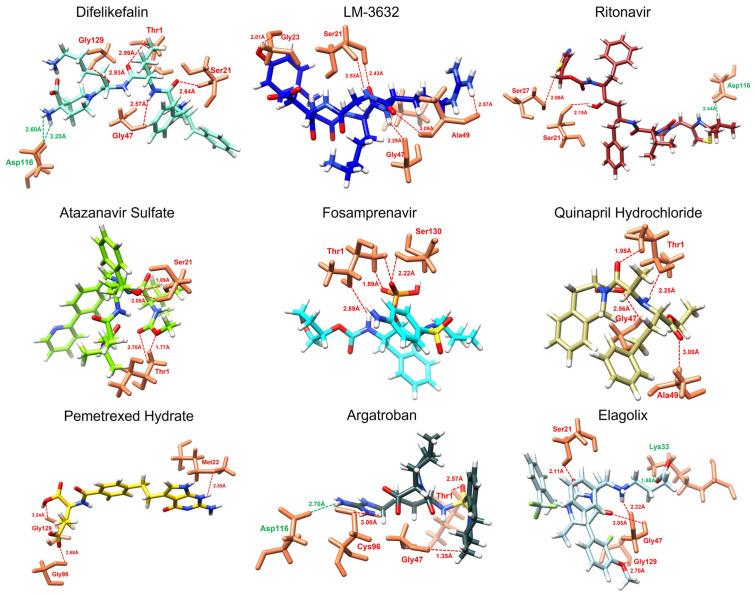
The figure represents the 3D interactions of the screened compounds against the malarial proteasome. Each ligand was represented in different colors while the color of the protein active site amino acid residues remained consistent.

Pemetrexed Hydrate interacted with Gly129, Met22, and Gly98 in the malarial proteasome with moderate bond distances of 3.24 Å, 2.85 Å, and 2.68 Å, while it showed fewer interactions with the human proteasome, mainly with Thr21, at a shorter bond distance of 1.97 Å. Argatroban formed strong bonds with Thr1, Cys96, and Gly47 in the malarial proteasome, with distances as short as 1.35 Å, while in the human proteasome, it showed comparable, yet slightly weaker, interactions. The presence of a salt bridge with Asp116 in the malarial proteasome suggests it might be a more selective inhibitor for malaria (Figure 2).

Finally, Elagolix Sodium interacted with Ser21, Gly129, Gly47 and Lys33, with bond distances ranging from 2.11 Å to 3.05 Å. Although it formed bonds with Lys33 and Gly23 in the human proteasome, the presence of a salt bridge suggests that further analysis is needed for this compound. The graphical depiction of the screened compounds against the human proteasome is shown in Supplementary Data Figure S1. Overall, the comparison indicates that several of these drugs show promising selectivity for the malarial proteasome based on their binding interactions and formation of stabilizing salt bridges. This selectivity could be crucial for developing malaria-specific proteasome inhibitors, minimizing off-target effects in human cells.

### 2.5. Molecular Dynamics Simulation

Molecular dynamics (MD) simulations provide insights into the dynamic behavior of protein–ligand interactions, revealing potential alterations in binding modes and the robustness of these interactions under physiological conditions. MD simulations also help in identifying any transient states that may enhance the inhibitory activity of compounds. To evaluate the stability of the identified compounds against TACE, the docked complexes were simulated for 100 nanoseconds using the GROMACS 2019.3 software.

#### 2.5.1. RMSD

**Figure 3:**
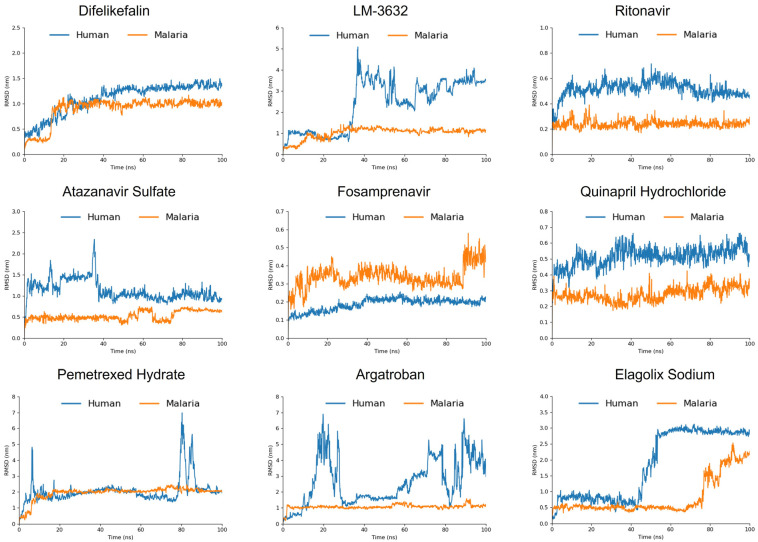
The RMSD results of the 100 ns MD simulation of the screened compounds against the malarial proteasome in comparison with the human proteasome.

The Root Mean Square Deviation (RMSD) analysis provided insightful results, highlighting the selective binding of most screened compounds to the malarial proteasome over the human proteasome. Among the compounds, LM-3632, Atazanavir Sulfate, Argatroban, and Pemetrexed Hydrate demonstrated high selectivity for the malarial proteasome, displaying stable RMSD profiles throughout the simulation, while their binding to the human proteasome was characterized by significant fluctuations, indicating weaker and less stable interactions. This aligns with the molecular docking results, where these compounds showed more favorable binding energies to the malarial proteasome, particularly LM-3632 and Atazanavir Sulfate reinforcing their potential as selective inhibitors (Figure 3). In contrast, Fosamprenavir was unique in exhibiting low RMSD values with the human proteasome, suggesting that it binds more stably to the human target.

Ritonavir and Quinapril Hydrochloride also emerged as promising candidates, showing lower RMSD values for the malarial proteasome, further supporting their potential as selective inhibitors, which is consistent with their relatively strong docking energies. Difelikefalin, which had the lowest docking energy, exhibited a slight preference for the malarial proteasome in the RMSD analysis, although it is not as pronounced as for some other compounds, suggesting moderate selectivity.

Interestingly, Elagolix Sodium showed stable RMSD values with the malarial proteasome for up to 75 ns, after which a sharp increase in the RMSD occurred, indicating ligand displacement. A similar shift in the RMSD was observed with the human proteasome but at an earlier time point (42 ns), suggesting that while Elagolix Sodium initially binds stably to the malarial proteasome, its selectivity diminishes over time. This behavior corresponds with its moderate docking energy and interaction profile, highlighting that although it shows some selectivity initially, its binding stability may be less reliable compared with other compounds.

#### 2.5.2. MD Interaction Energy

The interaction energy (IE) analysis, which includes Coulombic short-range (Coul-SR) and Lennard-Jones short-range (LJ-SR) contributions, provides further insights into the binding energies of the screened compounds for both the malarial and human proteasomes. The interaction energy graphs against the human proteasome are shown in Supplementary Data Figure S2. These results align with the RMSD data, highlighting selectivity toward the malarial proteasome for several compounds.

Fosamprenavir displayed the most favorable interaction energy with the malarial proteasome, totaling −489.916 KJ/mol, which was driven by strong Coul-SR (−373.208 KJ/mol) and LJ-SR (−116.708 KJ/mol) components. LM-3632 demonstrated a more balanced interaction profile, with a total energy of −355.736 KJ/mol for the malarial proteasome, which was significantly lower than its human counterpart (−187.083 KJ/mol). This selectivity is supported by the RMSD results, where LM-3632 exhibited stable binding to the malarial proteasome and fluctuating behavior with the human proteasome, reinforcing its potential as a selective inhibitor. Furthermore, Ritonavir showed a strong interaction with the malarial proteasome (−407.229 KJ/mol), in contrast to its high total energy for the human proteasome (−243.263 KJ/mol).

Atazanavir Sulfate exhibited a relatively modest total energy for the malarial proteasome (−229.562 KJ/mol), with strong contributions from both Coul-SR and LJ-SR forces. Its binding to the human proteasome, with a total energy of −158.878 KJ/mol, was significantly weaker, aligning with the RMSD results that showed stable binding to the malarial proteasome but more fluctuating behavior with the human proteasome (Table 3). Additionally, Quinapril Hydrochloride had a high interaction energy with the malarial proteasome (−414.713 KJ/mol), with strong Coul-SR (−333.538 KJ/mol) and moderate LJ-SR (−81.175 KJ/mol) components. In comparison, its binding to the human proteasome was substantially weaker (−181.223 KJ/mol), which agrees with its lower RMSD values for the malarial proteasome, suggesting good selectivity.

Pemetrexed Hydrate and Argatroban also demonstrated good and selective interaction energy profiles. Pemetrexed Hydrate had a high interaction energy with the malarial proteasome (−196.667 KJ/mol) compared with most compounds, although this was still considerably lower than its interaction with the human proteasome (−100.783 KJ/mol). Argatroban exhibited a notable difference between its total interaction energy for the malarial proteasome (−385.988 KJ/mol) and the human proteasome (−102.618 KJ/mol), suggesting potential selectivity.

**Figure 4:**
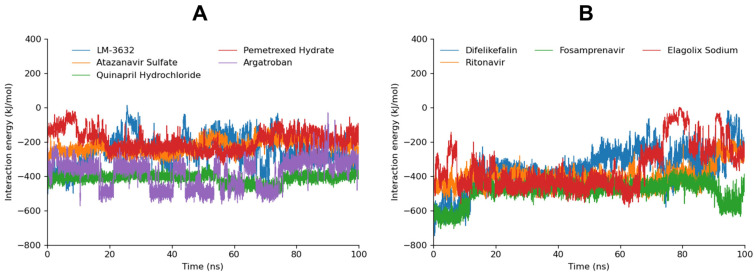
The MD interaction energy of the simulated compounds against the malarial proteasome. (**A**) represents the MD interaction energy of LM−3632, Quinapril Hydrochloride, Argatroban, Atazanavir Sulfate, and Pemetrexed Hydrate while (**B**) represents the MD interaction energy of Difelikefalin, Fosamprenavir, Elagolix Sodium, and Ritonavir.

Difelikefalin, while showing the lowest docking energy, had a total interaction energy of −355.373 KJ/mol for the malarial proteasome and −373.569 KJ/mol for the human proteasome, indicating less selectivity. Moreover, Elagolix Sodium also exhibited a relatively high total interaction energy for the malarial proteasome (−352.414 KJ/mol) compared with the human proteasome (−209.453 KJ/mol), which is consistent with the RMSD data showing stable binding for the initial portion of the simulation. However, its selectivity diminishes over time, as indicated by the increasing RMSD values for both proteasomes (Figure 4).

#### 2.5.3. Hydrogen Bonds

**Figure 5:**
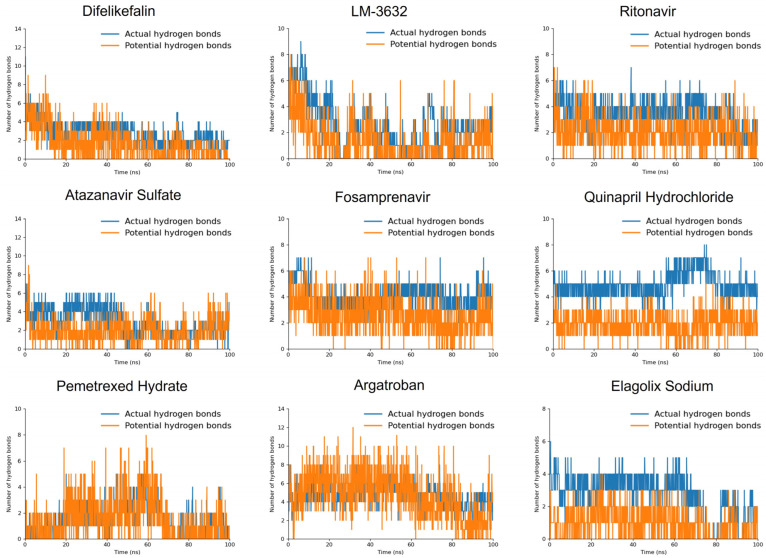
Hydrogen-bond patterns of the simulated compounds against the malarial proteasome throughout a 100 ns MD trajectory.

LM-3632, Ritonavir, Fosamprenavir, Quinapril Hydrochloride, and Argatroban maintained 1–2 stable hydrogen bonds throughout the entire MD trajectory, along with several potential hydrogen bonds. This stability correlates well with their strong interaction energy profiles and low RMSD values for the malarial proteasome, particularly for LM-3632 and Ritonavir, which showed high selectivity for the malarial target (Supplementary Data Figure S3) (Figure 5).

In contrast, Difelikefalin and Elagolix Sodium exhibited strong hydrogen-bonding profiles at the beginning of the molecular dynamics (MD) simulation, but both compounds showed a gradual decline in the number of actual and potential hydrogen bonds as the simulation progressed. This trend is consistent with their interaction energy and RMSD results, where Difelikefalin displayed similar binding energies for both the malarial and human proteasomes, while Elagolix Sodium showed initial stability in the malarial proteasome but eventually exhibited ligand displacement.

Pemetrexed Hydrate, although displaying fewer actual hydrogen bonds, still demonstrated a number of potential hydrogen bonds during the simulation. This aligns with its relatively moderate interaction energy and stable RMSD values for the malarial proteasome, suggesting that while it may not form as many immediate interactions, its binding remains stable over time. Furthermore, Atazanavir Sulfate also started with a strong hydrogen-bonding profile, although the number of potential hydrogen bonds decreased during the middle of the simulation, only to be restored at 80 ns. This behavior is consistent with its RMSD results, which showed fluctuations in its binding stability, and its moderate interaction energy with the malarial proteasome, indicating a potential for selectivity despite its intermediate stability.

#### 2.5.4. MD Snapshots at 100 ns

To validate our MD simulation results, snapshots were captured at 100 ns for compounds that demonstrated exceptional profiles in the previous analyses. Six compounds—Argatroban, Atazanavir Sulfate, LM-3632, Pemetrexed Hydrate, Quinapril Hydrochloride, and Ritonavir—were selected based on their RMSD values, hydrogen-bond stability, and MD interaction energy analysis. This refined screening allowed us to focus on the most promising candidates.

**Figure 6:**
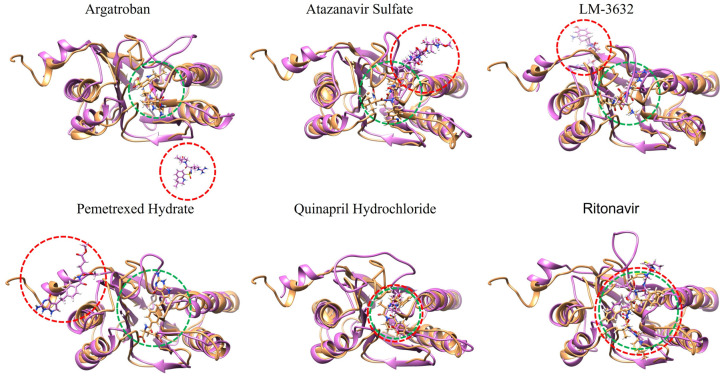
The 100 ns snapshot analysis of the screened compounds, superimposed with the human and malarial proteasomes, confirms the MD simulation results. The human proteasome is colored pink while the malarial proteasome is colored sandy brown. The doted circles denote the position of the ligand the red for the human-bound ligand and the green for the malarial proteasome β5 bound ligand.

The analysis, conducted using UCSF Chimera v1.16, revealed that all the selected compounds remained within the binding pocket of the malarial proteasome. Notably, Argatroban, LM-3632, and Pemetrexed Hydrate exited the binding pocket of the human proteasome, which correlates with their high selectivity and stable RMSD profiles for the malarial proteasome (Figure 6). In contrast, Atazanavir Sulfate underwent a slight conformational shift, moving partially out of the human proteasome’s binding pocket.

Quinapril Hydrochloride, which exhibited low interaction energy with the malarial proteasome in the MD interaction energy analysis, remained in the binding pockets of both the human and malarial proteasomes, indicating that it needs to be further examined. Similarly, Ritonavir stayed in the active sites of both proteasomes, which is consistent with its comparable interaction energy and RMSD values for both targets. These observations confirm that Argatroban, LM-3632, and Pemetrexed Hydrate stand out as the most selective compounds, showing strong potential for malarial proteasome inhibition with minimal activity on the human proteasome.

### 2.6. Binding Free-Energy Calculation

The whole trajectories of the 100 ns MD simulations were subjected to gmx_MMPBSA analysis to compute the binding free energy of the screened compounds. The gmx_MMPBSA tool was employed, and the MM/PBSA method was used to compute the binding energy with default parameters. The free-energy calculation enabled us to rank the final compounds based on their high binding affinity (Table 4).

Argatroban exhibited the most favorable binding affinity, with a ΔG of −32.17 kcal/mol, indicating a stable interaction with the target. Pemetrexed Hydrate followed, with a ΔG of −22.35 kcal/mol, which also suggest good binding affinity. Atazanavir Sulfate showed a ΔG of −18.35 kcal/mol, reflecting a moderate inhibition. A ΔG of −17.62 kJ/mol for LM-3632, while being the least favorable binding affinity among the screened compounds, suggested a stable yet slightly weaker interaction.

## 3. Discussion

The malarial proteasome has emerged as a promising target for antimalarial therapies due to its critical role in parasite viability across multiple life cycle stages, and its inhibition offers the potential to overcome the limitations associated with resistance to frontline drugs, including artemisinin-based therapies. This work builds on the growing interest in proteasome inhibitors for infectious diseases and leverages computational screening to refine the compound selection, prioritizing those with both high binding affinity to the malarial proteasome and favorable structural dynamics.

Our results revealed several compounds that demonstrated selective binding profiles. For instance, compounds such as Argatroban, LM-3632, Atazanavir Sulfate, and Pemetrexed Hydrate maintained favorable binding conformations within the malarial proteasome binding pocket, as indicated by stable MD simulation results. Additionally, the low binding energies and stable conformations of Quinapril Hydrochloride and Ritonavir highlighted their potential as selective inhibitors. However, the structural drift in Ritonavir and the position maintenance of Quinapril Hydrochloride within both the malarial and human proteasome pockets point to areas where optimization could further enhance selectivity.

Each of the resulting screened compounds in this study holds unique properties that have shown promise against the malarial proteasome. Argatroban, an anticoagulant primarily used in the management of thrombotic conditions, has not previously been investigated in the malarial proteasome [36,37,38]. However, its specific protease-inhibitory properties are intriguing given the sensitivity of the *P. falciparum* proteasome to functional disruptions [39]. Argatroban’s low docking and interaction energies in this study suggest a potentially valuable role as a selective inhibitor against the malarial proteasome. Moreover, Pemetrexed Hydrate, an antifolate chemotherapeutic used in oncology, has likewise not been traditionally associated with malaria treatment. However, the compound’s mechanism of action in disrupting nucleotide synthesis pathways has potential cross-applicability given the nucleotide reliance of malarial parasites, especially during asexual replication [40,41,42]. Previous studies have explored antifolate compounds like sulfadoxine–pyrimethamine as antimalarials, underscoring the potential for agents like Pemetrexed Hydrate to provide synergistic effects or new pathways for disrupting parasite survival [43,44].

Prior research has also shown that HIV protease inhibitors may have crossover effects against malaria given the structural similarities among some parasite proteases [45]. Although Atazanavir Sulfate’s effectiveness against malaria in vivo has not yet been fully tested, its molecular stability in the binding pocket of the malarial proteasome observed in this study aligns with findings from earlier research on protease-targeting drugs that hint at similar cross-inhibitory capabilities between retroviral and malarial proteases [46,47]. Furthermore, LM-3632 exhibited high specificity for the malarial proteasome as opposed to the human counterpart. This level of selectivity is promising, as selectivity is a major consideration in antimalarial therapy to avoid host toxicity. The compound’s high binding affinity for the malarial proteasome in molecular dynamics simulations adds to a growing body of evidence suggesting that optimized small molecules can selectively target the parasite proteasome, a target previously validated by studies on proteasome inhibitors against malaria.

This study’s findings contribute to the expanding pool of potential repurposed drugs for malaria and align with ongoing research that underscores the importance of selective proteasome inhibitors in overcoming resistance to standard treatments. By providing molecular dynamics and interaction profiles for these compounds, our work supports further investigation of these FDA-approved drugs as candidates for antimalarial therapy, reinforcing the relevance of proteasome inhibition in antimalarial drug design.

## 4. Materials and Methods

### 4.1. Pharmacophore Generation and Library Screening

The Receptor–Ligand Interaction Pharmacophore Generation (RLIPG) protocol in Discovery Studio generates pharmacophore models directly from receptor–ligand interactions. The RLIPG protocol has some notable features: (i) it is fully automated and quickly converts receptor–ligand complexes into pharmacophore models, (ii) it uses adjustable constraints to determine the receptor–ligand interactions, and (iii) it creates all possible pharmacophore combinations, ranks the pharmacophores by decreasing selectivity score, and returns the top-ranked ones.

An FDA-approved drug library was downloaded from an online vendor’s website (selleckchem.com (accessed on 10 July 2024)) and the Discovery Studio’s Library screen approach was applied to screen the pharmacophore-corresponding compounds.

### 4.2. Molecular Docking

Molecular docking is a well-established computational method used to predict the binding affinity between ligands and receptor proteins. It has become a powerful tool in drug discovery and development [48,49]. By determining the optimal alignment of a ligand within the binding site of a receptor, molecular docking estimates the interaction energy required for binding. This process involves the use of scoring functions to evaluate the strength and stability of these interactions, providing insight into the potential efficacy of drug candidates [50].

The molecular docking analysis was performed by targeting the β5 subunit of the malarial (*P. falciparum*) proteasome. The 3D structure of the proteasome was obtained from the RCSB Protein Data Bank using PDB ID: 7LXT. Prior to docking, all water molecules and other proteasome subunits were removed to avoid interference with the binding process. The binding pocket of the receptor was defined using the DBS (Define Binding Site) approach in Discovery Studio, which involved selecting the co-crystallized ligands to precisely generate the active site. After defining the pockets, the co-crystalized ligand was removed to enable molecular docking of the screened compounds.

Both the receptor and the screened compounds underwent minimization and preparation to optimize their structures before initializing molecular docking. For the receptor, hydrogen atoms were added, ensuring the correct protonation states and structural completeness of the protein. In the case of the ligands, the preparation included generating tautomers, adjusting ionization states to reflect biological conditions, and correcting any improper valencies. These tasks were accomplished through the Ligand Preparation module in Discovery Studio Client v22.

The human proteasome β5 subunit (PDB ID: 7LXV) was similarly prepared using the same protocol to enable a direct comparison with the malarial proteasome. This preparation was conducted to screen FDA-approved drugs for their potential to selectively inhibit the malarial proteasome while sparing the human counterpart. Therefore, molecular docking was carried out using the CDocker module in Discovery Studio, employing default settings for both orientation and conformation to maintain a standardized and consistent approach. Separate docking simulations were performed for the human and malarial proteasome receptors. The evaluation of the docked complexes was based on their docking energy values, measured in kcal/mol, which reflect the strength and stability of the interactions between the receptors and the screened compounds.

### 4.3. Molecular Dynamics Simulation

The molecular dynamics (MD) simulations in this study were performed for over 100 nanoseconds, following established methods for accuracy [51]. The compounds with the most favorable docking scores were selected for MD analysis. The CHARMM36 force field was employed, with the system setup performed via the CHARMM-GUI web interface to generate the necessary input files for the MD simulations. Furthermore, the system was solvated using the TIP3P water model, with periodic boundary conditions applied within a cubic simulation box. Counter ions were added to neutralize the system, and interactions were calculated using the Verlet method with a 10 Å cut-off radius. The LINCS algorithm constrained the bond lengths, while the Particle Mesh Ewald (PME) method ensured accurate electrostatic calculations. Energy minimization was performed using the steepest-descent method followed by two equilibration phases: one under constant volume and temperature (NVT), and the second under constant pressure and temperature (NPT). The detailed structural analysis of protein–ligand interactions was carried out using GROMACS 2019.3, with a 2 fs time step for stability during simulations

### 4.4. Free-Energy Calculation

The program gmx_MMPBSA v1.6.3 was designed to calculate the end-state free energies of protein–ligand complexes based on GROMACS molecular dynamics (MD) trajectory data [52]. Using the MM/PBSA method, binding free energies were estimated from MD trajectories in an explicit solvent by separately analyzing the complex, receptor, and ligand [53]. The binding free energy (ΔG_binding) of the lead compounds with the protein was calculated using the following equation:

ΔG_binding_ = G_complex_ − (G_protein_ + G_ligand_) (1)

Here, G_complex_ represents the energy of the protein–ligand complex, while G_protein_ and G_ligand_ denote the individual energies of the protein and ligand in aqueous environments, respectively.

## 5. Conclusions

This study provides a comprehensive analysis of FDA-approved compounds targeting the malarial proteasome, an emerging and highly specific drug target. Through a combination of pharmacophore modeling, molecular docking, and MD simulations we identified Argatroban, LM-3632, Atazanavir Sulfate, and Pemetrexed Hydrate as potential inhibitors with selective affinity toward the malarial proteasome over the human proteasome. Argatroban and Pemetrexed Hydrate demonstrated the lowest binding energy and selectivity, which can be attributed to their stable interactions within the binding pocket, which were confirmed by low RMSD fluctuations and comparatively favorable interaction energy profiles during the MD simulations. Structural optimization of Quinapril Hydrochloride and Ritonavir could present a promising avenue for enhancing their efficacy and selectivity as antimalarial agents. Both compounds showed a relatively strong binding affinity toward the malarial proteasome compared with the human proteasome; however, their interaction profiles suggest that there is room for improvement to maximize their antimalarial potential. Considering Quinapril Hydrochloride’s selectivity, it is also evident that exploring flavonoids for selective malarial drug targets could also be beneficial for future studies. These findings support further preclinical evaluations of these compounds and their potential for optimization as a new class of selective antimalarial therapies, which could help counteract the increasing resistance to current treatment regimens like artemisinin.

## Figures and Tables

**Table 1:** Molecular docking results of the screened compounds against both the malarial and human proteasomes.

FDA Compounds	Malaria		Human	
	CDocker Energy (kcal/mol)	CDocker Interaction Energy (kcal/mol)	CDocker Energy (kcal/mol)	CDocker Interaction Energy (kcal/mol)
Difelikefalin	−91.3408	−76.1250	−76.1867	−58.2198
LM−3632	−73.1960	−54.7689	−64.4805	−47.5619
Ritonavir	−67.7914	−64.0877	−61.2586	−60.8960
Atazanavir Sulfate	−64.1737	−66.8728	−58.6843	−53.5790
Fosamprenavir	−59.9645	−56.3789	−56.2256	−58.3490
Quinapril Hydrochloride	−47.5670	−52.5940	−43.8536	−50.3382
Zofenopril Calcium	−44.3744	−58.5426	−36.7981	−60.4126
Pemetrexed Hydrate	−44.2371	−48.5265	−42.6123	−46.3788
Argatroban	−43.6825	−46.2094	−40.4534	−44.7932
Elagolix Sodium	−39.4056	−63.2939	−24.1487	−52.4867
Alvimopan	−38.0832	−52.8094	−37.0859	−54.3181
Febantel	−35.9661	−43.0001	−34.5864	−41.7772
Gabexate Mesylate	−35.3583	−42.1645	−31.8792	−38.3130
Amprenavir	−34.7707	−48.1583	−30.1229	−46.4217
Ramatroban	−34.3151	−45.3638	−32.8876	−45.0298

**Table 2:** Binding interactions of the screened compounds to the malarial proteasome in comparison with the human proteasome. The Bold letters represents the salt bridges formed during the analysis.

Drugs	Malaria		Human	
	Amino Acids	Bond Distance	Amino Acids	Bond Distance
Difelikefalin	Gly129, Thr1 Ser21, Gly47 **Asp116**	2.93 Å, 2.99 Å 2.64 Å, 2.57 Å **2.60 Å, 2.20 Å**	Thr21 Gly47	2.58 Å, 2.18 Å 2.01 Å
LM-3632	Gly23, Gly47 Ser21 Ala49	2.01 Å, 2.29 Å 2.53 Å, 2.43 Å 3.09 Å, 2.57 Å	Gly23 Thr21 Gly47, Met45	2.39 Å 2.98 Å, 1.99 Å 2.45 Å, 2.29 Å
Ritonavir	Ser27, Ser21 **Asp116**	2.98 Å, 2.15 Å **2.44 Å**	Thr21, Gly47 Gly129	2.98 Å, 1.94 Å 2.77 Å
Atazanavir Sulfate	Ser21 Thr1	1.89 Å, 2.08 Å 2.70 Å, 1.77 Å	Thr21, Lys33 Thr1, Ala49	2.98 Å, 2.68 Å 2.62 Å, 3.05 Å
Fosamprenavir	Thr1 Ser130	2.89 Å, 2.22 Å, 2.89 Å 2.96 Å	Thr21, Thr1 Gly47	2.68 Å, 2.86 Å 1.94 Å
Quinapril Hydrochloride	Thr1 Gly47, Ala49	1.98 Å, 2.25 Å 2.56 Å, 3.00 Å	Gly23, Thr21 Lys33, Thr1 Gly47	2.36 Å, 2.92 Å 2.04 Å, 2.11 Å 2.06 Å
Pemetrexed Hydrate	Gly129, Gly98 Met22	3.24 Å, 2.68 Å 2.85 Å	Thr21	1.97 Å
Argatroban	Thr1, Gly47 Cys96, **Asp116**	2.57 Å, 1.35 Å 3.00 Å, **2.70 Å**	Gly23, Ala49 Thr21	2.66 Å, 2.74 Å 2.02 Å, 3.09 Å
Elagolix Sodium	Ser21, Gly129 Gly47 Lys33	2.11 Å, 2.70 Å 3.05 Å, 2.22 Å 2.88 Å	**Lys33** Thr21, Gly23	**1.97 Å** 2.33 Å, 2.51 Å

**Table 3:** The calculated interaction energy of the simulated compounds against the malarial proteasome in comparison with the human proteasome.

Compound	Malaria IE (KJ/mol)			Human IE (KJ/mol)		
	Coul-SR	LJ-SR	Total Energy	Coul-SR	LJ-SR	Total Energy
Difelikefalin	−264.769	−90.604	−355.373	−268.127	−105.442	−373.569
LM−3632	−163.274	−192.462	−355.736	−145.920	−41.163	−187.083
Ritonavir	−224.958	−182.271	−407.229	−55.031	−188.232	−243.263
Atazanavir Sulfate	−78.201	−151.361	−229.562	−38.883	−119.995	−158.878
Fosamprenavir	−373.208	−116.708	−489.916	−268.127	−105.442	−373.569
Quinapril Hydrochloride	−333.538	−81.175	−414.713	−52.053	−129.170	−181.223
Pemetrexed Hydrate	−94.442	−102.225	−196.667	−27.617	−73.166	−100.783
Argatroban	−284.080	−101.908	−385.988	−72.440	−30.178	−102.618
Elagolix Sodium	−235.416	−116.998	−352.414	−133.444	−76.009	−209.453

**Table 4:** The calculated binding free energy of four highly selective compounds.

Sr	Compounds	ΔG_(TOTAL)_	Standard Deviation
1	Argatroban	−32.17	7.01
2	Pemetrexed Hydrate	−22.35	7.35
3	Atazanavir Sulfate	−18.35	5.25
4	LM-3632	−17.62	7.10

## Data Availability

The data that support the findings of this study are available from the corresponding author upon reasonable request.

## Supplementary Materials

The following supporting information can be downloaded at: https://www.mdpi.com/article/10.3390/ijms252211881/s1.
