# Supplementary material for: Identification of Malaria-Selective Proteasome β5 Inhibitors Through Pharmacophore Modeling, Molecular Docking, and Molecular Dynamics Simulation
Source: Int J Mol Sci. 2024 Nov 5;25(22):11881. doi: 10.3390/ijms252211881 (PMC11593624; doi:10.3390/ijms252211881)
Supplement: Supplementary file 1 [file ijms-25-11881-s001.zip › ijms-3271897-supplementary.pdf]

**Supplementary Data**

# Identification of Malaria-Selective Proteasome β5 Inhibitors Through Pharmacophore Modeling, Molecular Docking, and Molecular Dynamics Simulation

**Muhammad Yasir [1], Jinyoung Park [1], Eun-Taek Han [2], Jin-Hee Han [2], Won Sun Park [3] and Wanjoo Chun [1,*]**

[1] Department of Pharmacology, Kangwon National University School of Medicine, Chuncheon 24341, Republic of Korea; yasir.khokhar1999@gmail.com (M.Y.); jinyoung0326@kangwon.ac.kr (J.P.)

[2] Department of Medical Environmental Biology and Tropical Medicine, Kangwon National University School of Medicine, Chuncheon 24341, Republic of Korea; ethan@kangwon.ac.kr (E.-T.H.); han.han@kangwon.ac.kr (J.-H.H.)

[3] Department of Physiology, Kangwon National University School of Medicine, Chuncheon 24341, Republic of Korea; parkws@kangwon.ac.kr

* Correspondence: wchun@kangwon.ac.kr; Tel.: +82-33-250-8853

**Table S1.** Docking energies of all 47 compounds manifested lower binding energy to the malarial proteasome.

| Sr No | FDA Compounds | Malaria | | Human | |
|---|---|---|---|---|---|
| | | CDocker Energy | CDocker Interaction Energy | CDocker Energy | CDocker Interaction Energy |
| 1 | Difelikefalin | -91.3408 | -76.1250 | -76.1867 | -58.2198 |
| 2 | LM-3632 | -73.1960 | -54.7689 | -64.4805 | -47.5619 |
| 4 | Ritonavir | -67.7914 | -64.0877 | -61.2586 | -60.8960 |
| 5 | Atazanavir Sulfate | -64.1737 | -66.8728 | -58.6843 | -53.5790 |
| 6 | Fosamprenavir | -59.9645 | -56.3789 | -56.2256 | -58.3490 |
| 7 | Quinapril hydrochloride | -47.5670 | -52.5940 | -43.8536 | -50.3382 |
| 8 | Zofenopril calcium | -44.3744 | -58.5426 | -36.7981 | -60.4126 |
| 9 | Pemetrexed hydrate | -44.2371 | -48.5265 | -42.6123 | -46.3788 |
| 10 | Argatroban | -43.6825 | -46.2094 | -40.4534 | -44.7932 |
| 11 | Elagolix Sodium | -39.4056 | -63.2939 | -24.1487 | -52.4867 |
| 12 | Alvimopan | -38.0832 | -52.8094 | -37.0859 | -54.3181 |
| 13 | Febantel | -35.9661 | -43.0001 | -34.5864 | -41.7772 |
| 14 | Gabexate mesylate | -35.3583 | -42.1645 | -31.8792 | -38.3130 |
| 15 | Aamprenavir | -34.7707 | -48.1583 | -30.1229 | -46.4217 |
| 16 | Ramatroban | -34.3151 | -45.3638 | -32.8876 | -45.0298 |
| 17 | Nizatidine | -28.7784 | -39.2043 | -25.3137 | -34.6601 |

| | | | | | |
|---|---|---|---|---|---|
| 18 | Dronedarone hydrochloride | -28.1877 | -49.3573 | -23.4569 | -44.3410 |
| 19 | Untitled (Ligand-633) | -28.1760 | -52.7643 | -22.7990 | -50.2239 |
| 20 | Cefuroxime axetil | -27.3677 | -47.5070 | -24.8376 | -47.6071 |
| 21 | Lapatinib Ditosylate | -26.6398 | -51.6110 | -24.6513 | -46.3819 |
| 22 | Verbascoside | -25.4876 | -53.5206 | -18.0412 | -53.9952 |
| 23 | Alogliptin | -25.3014 | -30.5759 | -22.4431 | -34.4925 |
| 24 | Nelfinavir mesylate | -25.0866 | -49.2870 | -15.9217 | -45.6617 |
| 25 | Elvitegravir | -25.0597 | -47.0061 | -22.6053 | -43.9505 |
| 26 | Mirodenafil | -23.1149 | -46.1572 | -22.8240 | -45.2303 |
| 27 | Rosuvastatin calcium | -21.4888 | -48.5616 | -20.9537 | -47.4544 |
| 28 | Eslicarbazepine Acetate | -20.9279 | -29.5108 | -19.7806 | -27.9669 |
| 29 | GSK2118436 | -20.3047 | -41.2678 | -16.9022 | -44.6646 |
| 30 | Primaquine diphosphate | -19.5287 | -34.5758 | -17.4685 | -32.2285 |
| 31 | Proadifen hydrochloride | -19.4740 | -29.6556 | -15.3471 | -33.5434 |
| 32 | Acemetacin | -18.1711 | -45.2331 | -15.5170 | -44.9046 |
| 33 | Fosaprepitant dimeglumine | -17.2855 | -55.5682 | -15.5237 | -48.5602 |
| 35 | Aztreonam | -12.1548 | -53.9945 | -11.7064 | -47.6228 |
| 36 | Cefetamet pivoxil hydrochloride | -11.8265 | -50.2039 | -9.2984 | -42.5399 |
| 37 | Asunaprevir | -10.7952 | -53.1559 | -5.2190 | -47.3741 |
| 38 | Atorvastatin calcium | -10.6278 | -56.6530 | -4.5720 | -55.2647 |
| 39 | Dynapen | -9.8703 | -38.7773 | -7.7473 | -34.7013 |
| 40 | Pirenzepine | -8.9048 | -36.2164 | -6.1036 | -38.7280 |
| 41 | Revefenacin | -8.2500 | -55.1834 | -5.4064 | -53.3332 |
| 42 | Benicar | -7.6451 | -51.2832 | -4.3707 | -48.5497 |
| 43 | Aprepitant | -7.2484 | -36.1075 | -6.2667 | -38.3139 |
| 44 | Efonidipine | -6.4447 | -45.3285 | -4.9149 | -50.4395 |
| 45 | Anlotinib | -6.4040 | -46.7386 | -1.9602 | -38.3186 |
| 46 | Ceftiofur hydrochloride | -6.1279 | -47.3532 | -3.8088 | -47.8099 |
| 47 | Yohimbine hydrochloride | -4.3229 | -34.4422 | -2.5051 | -38.4045 |

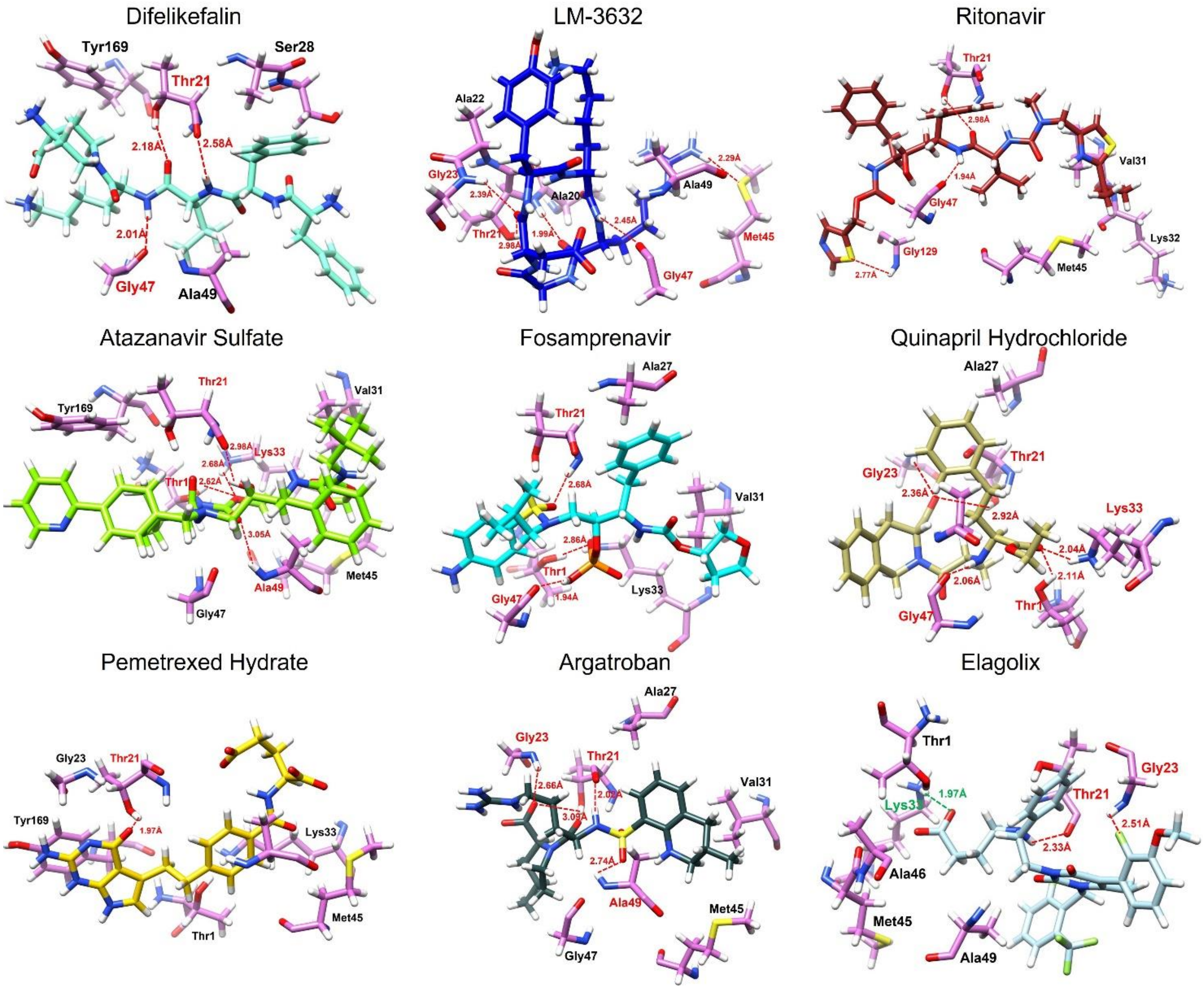


**Figure S1.** The interaction analysis of top 15 compounds against human proteasome. The hydrogen bond interactions are depicted in red while the salt bridges are depicted in green.

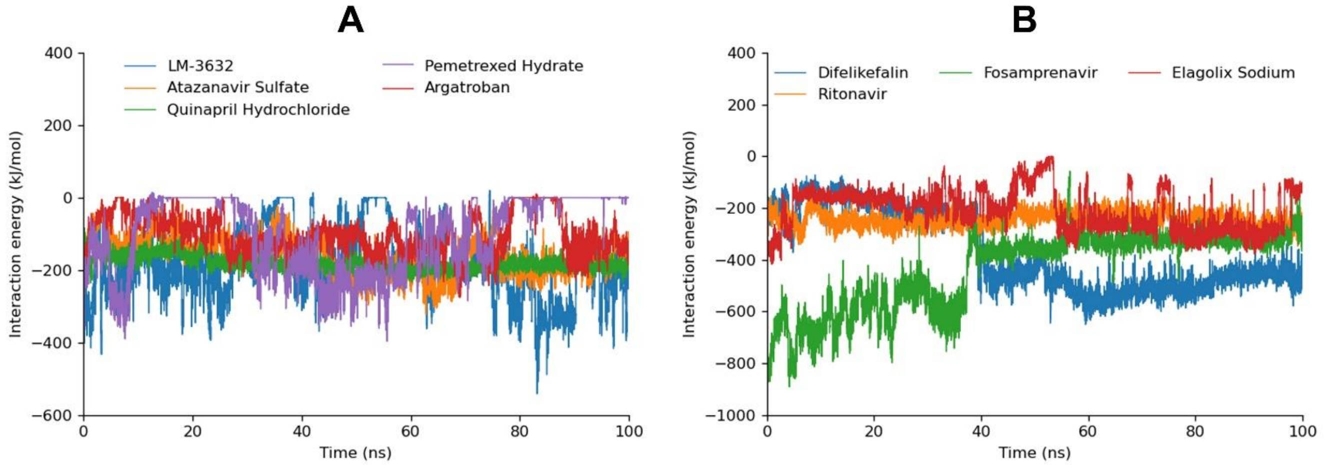


**Figure S2.** The interaction energy trends of screened compounds against human proteasome.

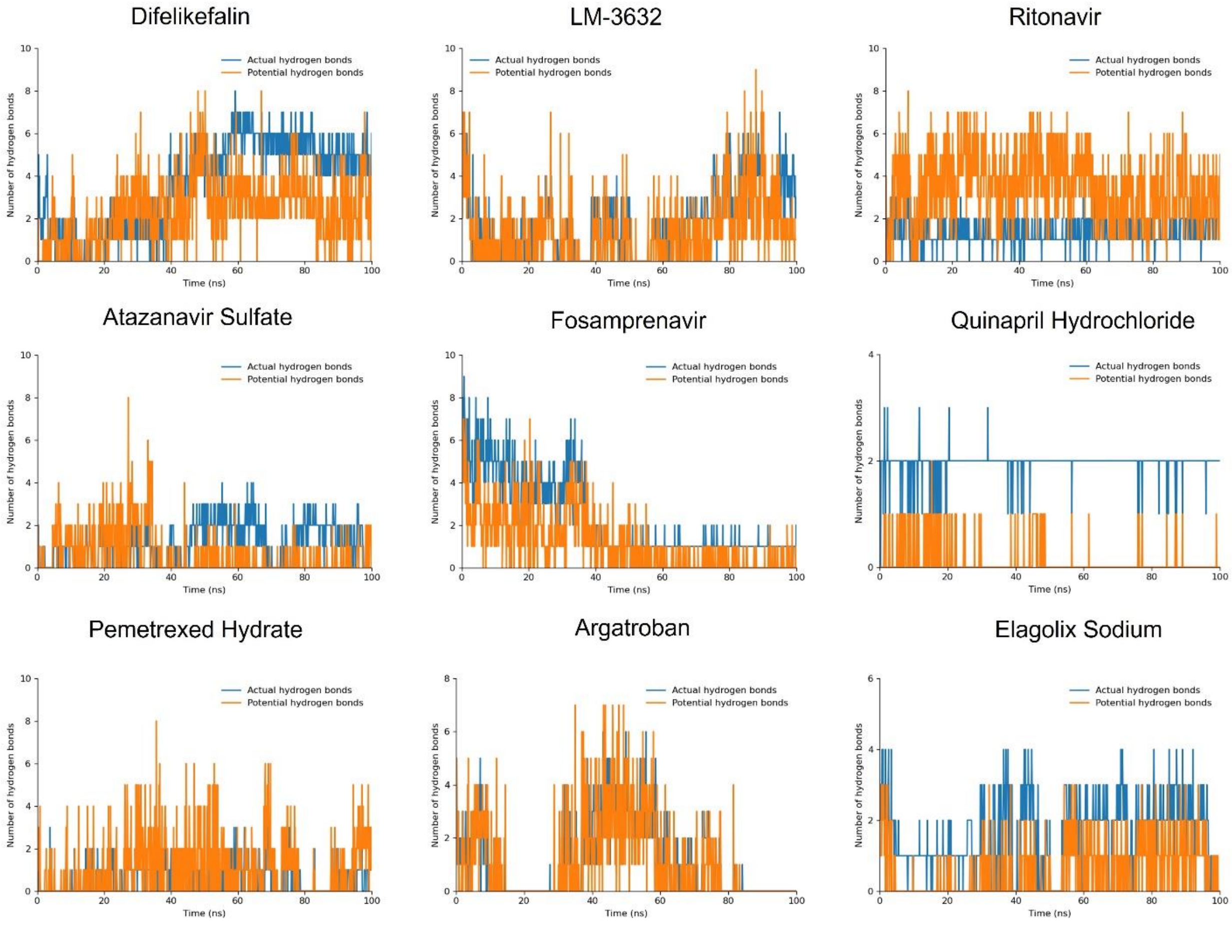


**Figure S3.** The hydrogen bond plots of screened compounds against human proteasome
